# McConnell’s Sign Still Holds Its Value: A Lesson Learned from Two Cases

**DOI:** 10.7759/cureus.6240

**Published:** 2019-11-26

**Authors:** Tikal Kansara, Fernando Quesada, Hansang Park, Kuldeep Ghosh, Mohammad Saeed

**Affiliations:** 1 Internal Medicine, New York Medical College - Metropolitan Hospital Center, New York, USA

**Keywords:** pulmonary embolism, mcconnell's sign, transthoracic echocardiography

## Abstract

Acute pulmonary embolism (PE) can be potentially fatal if not diagnosed and treated early. Mortality in untreated cases can be as high as 30%. Atypical presentation and submassive PE can be missed due to subtle clinical features. Computerized tomography pulmonary angiogram is expensive, exposes to radiation and carries the risk of contrast nephropathy or anaphylactic reactions. On the contrary, McConnell's sign, which is a highly specific sign of PE, can be demonstrated at the bedside with a transthoracic echocardiogram (TTE). Here we discuss two cases where bedside TTE demonstrating McConnell's sign helped in the diagnosis and treatment of PE.

## Introduction

The diagnosis of submassive to massive pulmonary embolism (PE) is critical to prevent mortality. In an emergent situation, computerized tomography (CT) pulmonary angiogram is the diagnostic modality of choice. In conditions like renal impairment or contrast allergy, the test cannot be performed. Ventilation-perfusion scan results may not be readily available. In pregnant patients with low pretest probability, these tests are not recommended. McConnell's sign is a regional right ventricular (RV) wall motion abnormality with hypokinesia of mid and basal walls sparing the RV apex [1]. A meta-analysis of the use of transthoracic echocardiogram (TTE) in diagnosing PE showed the sensitivity of McConnell's sign to be only 22% but high specificity of 97% [2]. In questionable cases, an inexpensive test like bedside echocardiogram demonstrating McConnell's sign can guide further investigations and treatment.

## Case presentation

Case I

**Figure 1:**
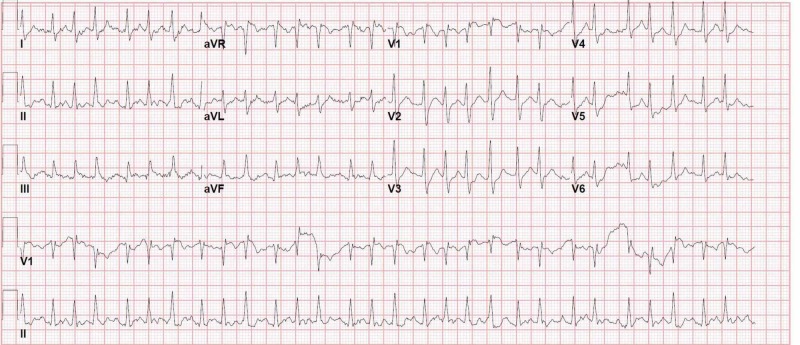
Initial ECG of case 1 showing atrial fibrillation with a rapid ventricular response of 174/min ECG, electrocardiogram

A 48-year-old man was brought to the emergency department (ED) for syncope. He also complained of shortness of breath on exertion for three days. On examination, he had tachycardia 177/min, tachypnea 24/min, normal blood pressure and oxygen saturation. Distended neck veins were noted on examination. The electrocardiogram (ECG) showed atrial fibrillation with a rapid ventricular response of 174/min (Figure 1).

Chest X-ray was normal. Basic laboratory investigations were significant only for mildly elevated creatinine of 1.4 mg/dl. A diagnosis of paroxysmal atrial fibrillation was made. He was given a stat dose of amiodarone and started on amiodarone drip with a significant reduction in heart rate to 110/min.

A quick bedside TTE showed severely dilated right atrium, moderately dilated RV, moderate tricuspid regurgitation, hypokinesia of basal and mid-RV free wall and normal contraction of the RV apex (McConnell's sign) (Video 1).

**Video 1:** Video showing akinesia of right ventricular mid and basal free and septal walls with normal contraction of apex suggestive of McConnell sign.

**Figure 2:**
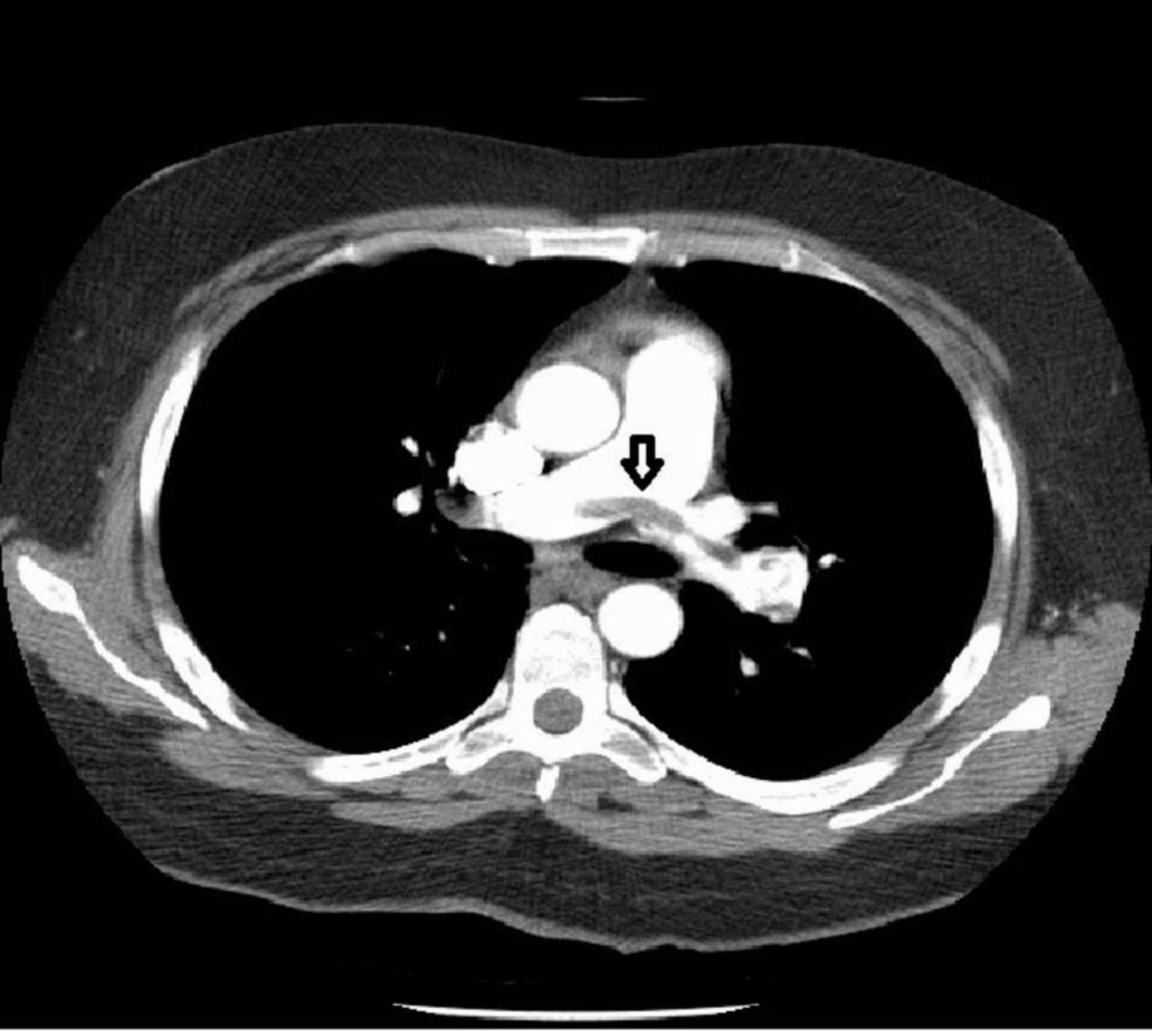
CT pulmonary angiogram showing saddle embolus in the main pulmonary artery

**Figure 3:**
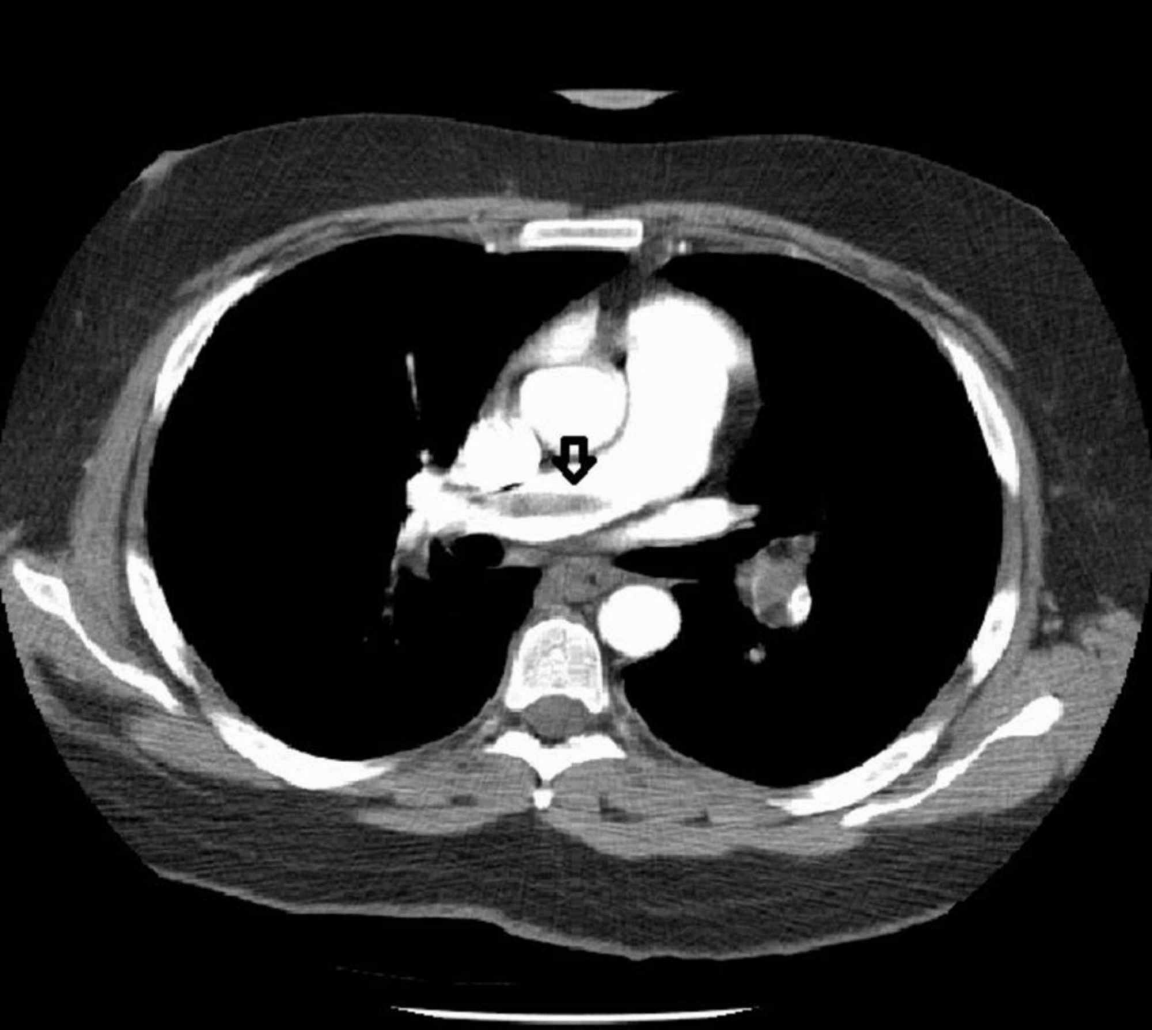
CT pulmonary angiogram showing embolus extending into the right main trunk

D-dimer was equivocal. The Pulmonary Embolism Rule-out Criteria (PERC) rule score was 1 (cannot rule out PE) and the Wells score was 1.5 (low-risk group; 1.3% of PE). Even though the Wells score was low, CT pulmonary angiogram was planned because of McConnell's sign on echocardiography. CT pulmonary angiogram was suggestive of a saddle pulmonary embolus within the main pulmonary artery (Figure 2) extending into the right and left pulmonary arteries (Figure 3). There was near-complete occlusion of the right and left secondary branches of pulmonary arteries.

**Figure 4:**
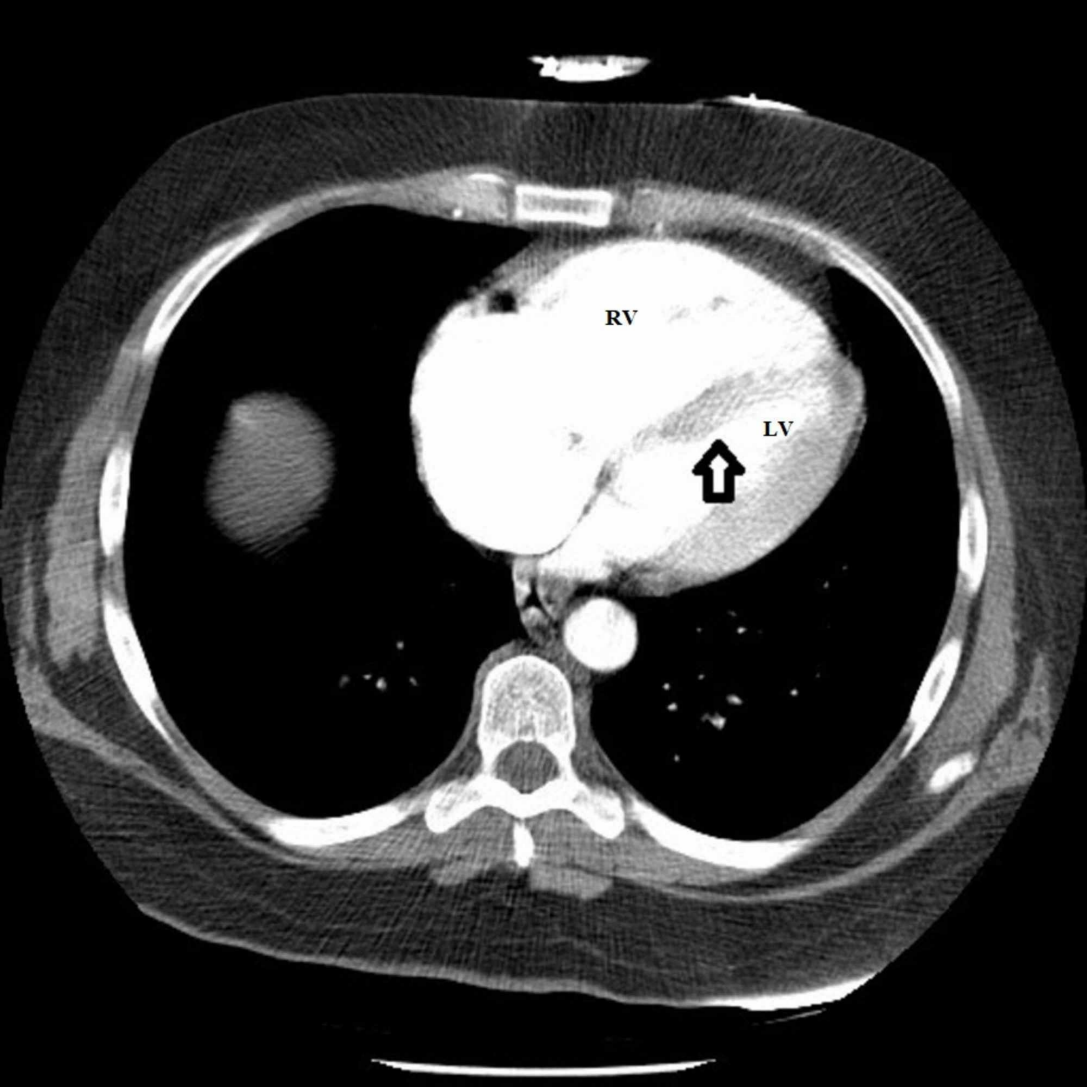
CT pulmonary angiogram showing flattening of the interventricular septum (arrow) with an increase in the size of the right ventricle (RV) and reduced left ventricular (LV) size

There was also flattening of the interventricular septum, decreased left-sided ventricular volume and increased RV volume compatible with the RV strain (Figure 4).

 

**Figure 5:**
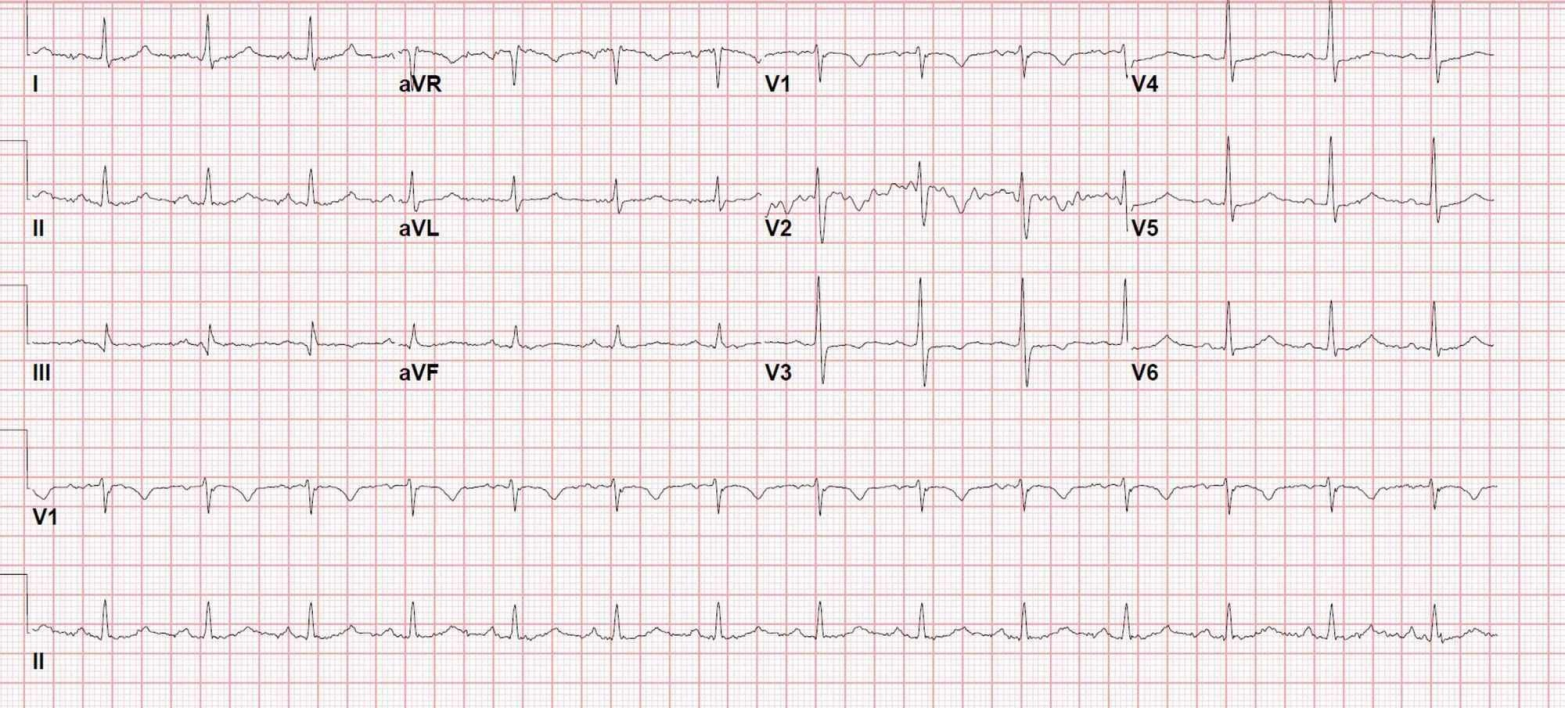
Normal ECG after 48 hours of treatment ECG, electrocardiogram

A diagnosis of submassive PE was made (as the patient had normal blood pressure). The HAS-BLED score was 0. He was anticoagulated with heparin and switched to oral anticoagulation. Repeat ECG after 48 hours showed normal sinus rhythm (Figure 5).

 

Without the bedside echocardiogram showing McConnell’s sign, a grave diagnosis of submassive PE would have been missed or delayed.

 

Case II

An 80-year-old woman was evaluated in ED for syncope that occurred while she was trying to stand up from sitting position. She denied chest pain, palpitation, headache, convulsion or incontinence. Her medical history was suggestive of hypertension, diabetes mellitus type 2, asthma and obesity grade 3. On examination, she had tachycardia 140/min, tachypnea 22/min and a normal blood pressure of 134/78 mmHg. Physical examination showed mildly tender and swollen left leg compared to the right. Duplex ultrasound of bilateral lower extremities showed acute deep vein thrombosis in common femoral vein, bilateral femoral and popliteal veins.

**Figure 6:**
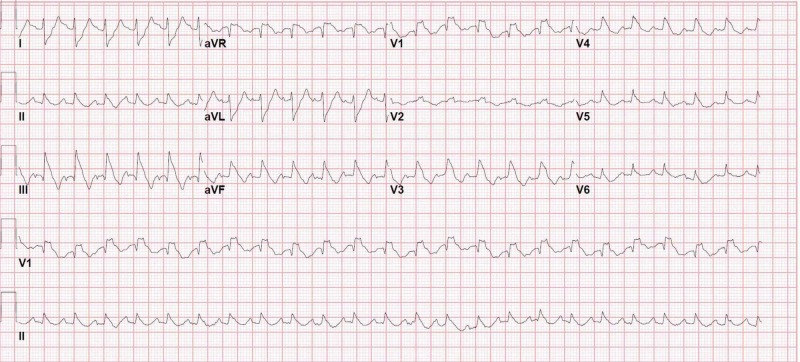
Initial ECG of case 2 showing new-onset right bundle branch block ECG, electrocardiogram

Laboratory investigations were significant for elevated creatinine of 2.4 mg/dl (patient’s baseline creatinine 1.1 mg/dl) and elevated troponin I 0.92 ng/ml. ECG was suggestive of sinus tachycardia with a new-onset right bundle branch block (Figure 6). The patient was advised urgent coronary angiogram but she refused and declined any other invasive intervention.

 

The Wells score was 7.5 (high-risk group; 40.6% chance of PE), the PERC rule score was 3 (cannot rule out PE). CT pulmonary angiogram was deferred in lieu of the elevated creatinine. Instead, a bedside TTE was done. It showed normal ejection fraction (EF > 55%), mildly dilated RV with reduced RVEF, elevated RV systolic pressure (>60 mmHg) and moderate tricuspid regurgitation. Hyperkinesis of the RV apex and hypokinesis of the RV free wall were also noted (McConnell's sign) (Video 2).

**Video 2:** Video showing hyperkinesis of right ventricular apex and hypokinesis of the right ventricular free wall (McConnell sign)

**Figure 7:**
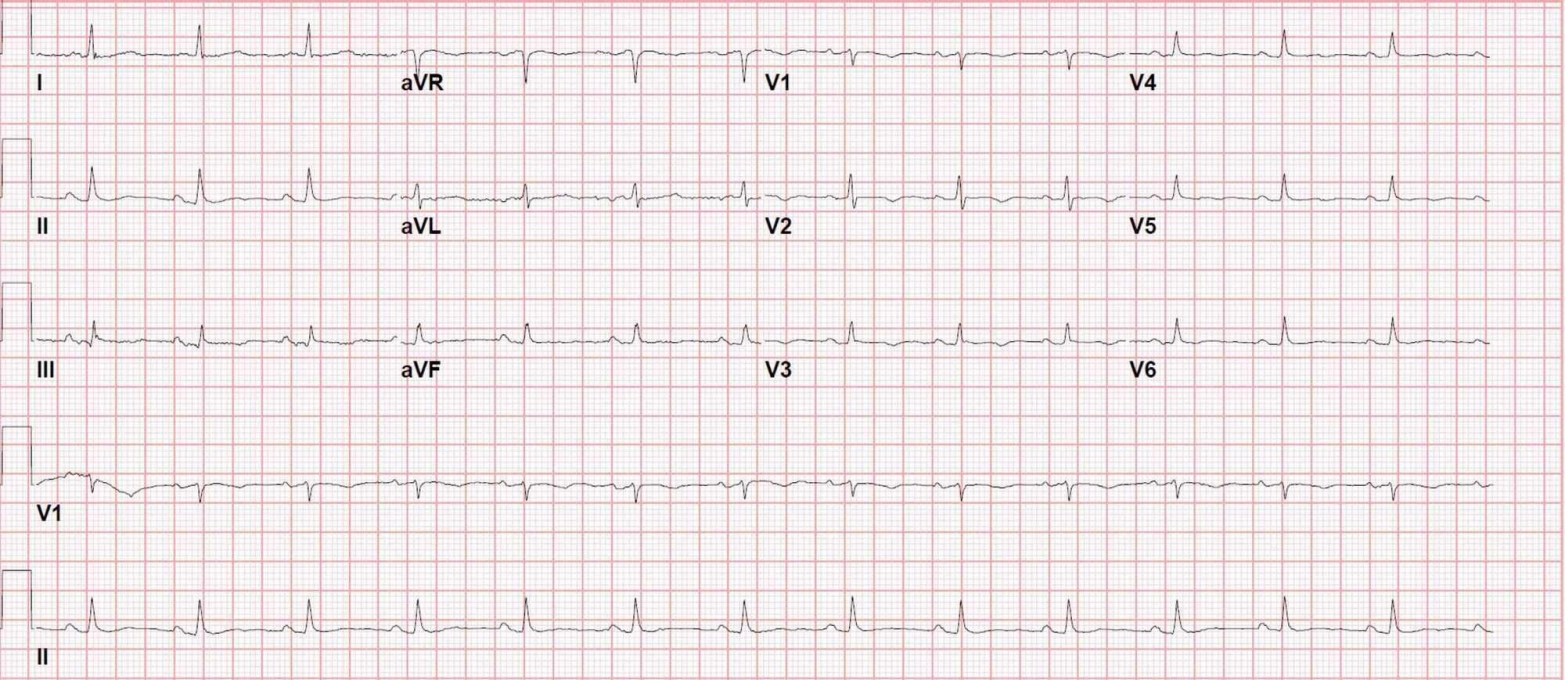
Pre discharge normal ECG of case 2 ECG, electrocardiogram

With a high suspicion of PE and McConnell’s sign on echocardiogram, she was started on heparin infusion and later shifted to oral anticoagulation. After a week, the patient was stable to be discharged. Repeat ECG before discharge was normal (Figure 7).

 

McConnell's sign along with the high Wells score justified starting and continuing anticoagulation to complete six months of therapy. 

## Discussion

The above-mentioned cases demonstrate the importance of McConnell's sign in PE. In normotensive patients, like the above two cases, TTE is not recommended for diagnosis but can be used for risk stratification [3]. In the situation where CT pulmonary angiogram is not feasible, a demonstration of RV dysfunction can justify the need for thrombolysis. Demonstrating McConnell's sign [1] can strongly suggest PE even if the clinical findings are non-specific.

McConnell et al. [1] in their original study found this distinct pattern of RV dysfunction at mid-free wall vs normal to be highly specific for acute PE (p = 0.0001). The study showed the positive predictive value of 77% and the negative predictive value of 96%. Over time, other studies have validated the results and came to a similar conclusion.

Bedside TTE is not just limited to the McConnell sign in PE. There are studies showing the importance of quantitative parameters of RV function, such as tricuspid annular plane systolic excursion, RV/left ventricular ratio and RV global and free wall longitudinal strain [4] in diagnosing PE. A study by Kearon et al. [5] showed that about 50% of diagnosed PEs are associated with RV dysfunction, which is associated with approximately fivefold greater in-hospital mortality.

## Conclusions

PE carries a high risk of mortality if left untreated. When present, McConnell's sign acts as a valuable marker for PE diagnosis. As McConnell's sign has a high specificity, its presence invariably rules in PE. Demonstrating McConnell's sign is a cheaper and risk-free alternative to CT pulmonary angiogram, although not a complete substitute. The importance of McConnell's sign is higher in patients where CT pulmonary angiogram is contraindicated and in subclinical cases, wherein McConnell's sign can act as a bridge and guide further investigations. Based on the above-mentioned points, it is worthwhile to perform TTE in patients suspected of PE where CT pulmonary angiogram is contraindicated. 
